# Calcite Kinetics for Spiral Growth and Two-Dimensional Nucleation

**DOI:** 10.1021/acs.cgd.2c00378

**Published:** 2022-05-30

**Authors:** Robert Darkins, Yi-Yeoun Kim, David C. Green, Alexander Broad, Dorothy M. Duffy, Fiona C. Meldrum, Ian J. Ford

**Affiliations:** † London Centre for Nanotechnology, University College London, 17-19 Gordon Street, London WC1H 0AH, UK; ‡ School of Chemistry, University of Leeds, Woodhouse Lane, Leeds LS2 9JT, UK

## Abstract

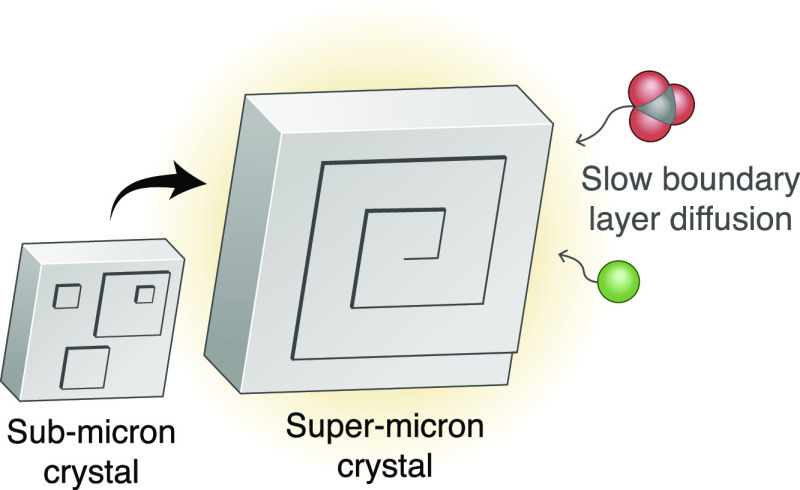

Calcite crystals grow by means of molecular steps that develop on {10.4} faces. These steps can arise stochastically via two-dimensional (2D) nucleation or emerge steadily from dislocations to form spiral hillocks. Here, we determine the kinetics of these two growth mechanisms as a function of supersaturation. We show that calcite crystals larger than ∼1 μm favor spiral growth over 2D nucleation, irrespective of the supersaturation. Spirals prevail beyond this length scale because slow boundary layer diffusion creates a low surface supersaturation that favors the spiral mechanism. Sub-micron crystals favor 2D nucleation at high supersaturations, although diffusion can still limit the growth of nanoscopic crystals. Additives can change the dominant mechanism by impeding spiral growth or by directly promoting 2D nucleation.

## Abstract 2

The growth kinetics of calcite are determined for both spiral growth and 2D nucleation as a function of the surface supersaturation. These kinetics reveal the dominant growth mechanism and the role of solute diffusion in single crystal growth.

## 1 Introduction

Calcite (CaCO_3_) is used as a model system to study how additives control crystallization kinetics and incorporate into crystals.^1−11^ Generally, these studies aim to understand either how biological organisms use additives to tailor the properties of biominerals or how we can use similar strategies to create new bio-inspired composites. A model that could predict the growth kinetics of calcite over a wide range of conditions could help to interpret these experimental studies and therefore help to design new strategies for growth inhibition and additive incorporation.

An obstacle in developing such a model is measuring the surface-controlled kinetics of calcite growth. The preeminent method for probing crystal growth processes in solution is flow-through atomic force microscopy (AFM). This method has been used extensively to study how supersaturation,^12−14^ stoichiometry,^15−17^ pH,^16^ and additives^18−20^ affect the growth of calcite. It had long been believed on the basis of variable flow rate tests that calcite growth was limited by surface reaction during flow-through AFM.^21^ It is now known, to the contrary, that variable flow rate tests are inconclusive and that the bulk supersaturation does not extend all the way to the surface of calcite under typical flow rates.^22^ Nevertheless, for AFM studies that report both the step velocities and the step densities of spiral hillocks, it remains possible to recover the surface-controlled kinetics using theory. Specifically, the step velocities and the step densities can be used to compute the critical step length, which provides an indicator of the surface supersaturation and therefore a means of characterizing mass transport in the experiment.^22^

In this paper, we determine the surface-controlled kinetics of calcite for both spiral growth and two-dimensional (2D) nucleation by applying the critical step length indicator^22^ to past AFM data.^12^ We relate our results to real-world crystallization by examining two issues: (i) how the mechanism (spiral growth versus 2D nucleation) and the limiting kinetics (reaction versus diffusion) depend on crystal size and bulk supersaturation and (ii) how additives might change the mechanisms and kinetics.

## 2 Results and Discussion

### 2.1 Surface-Controlled Kinetics

**Figure 1:**
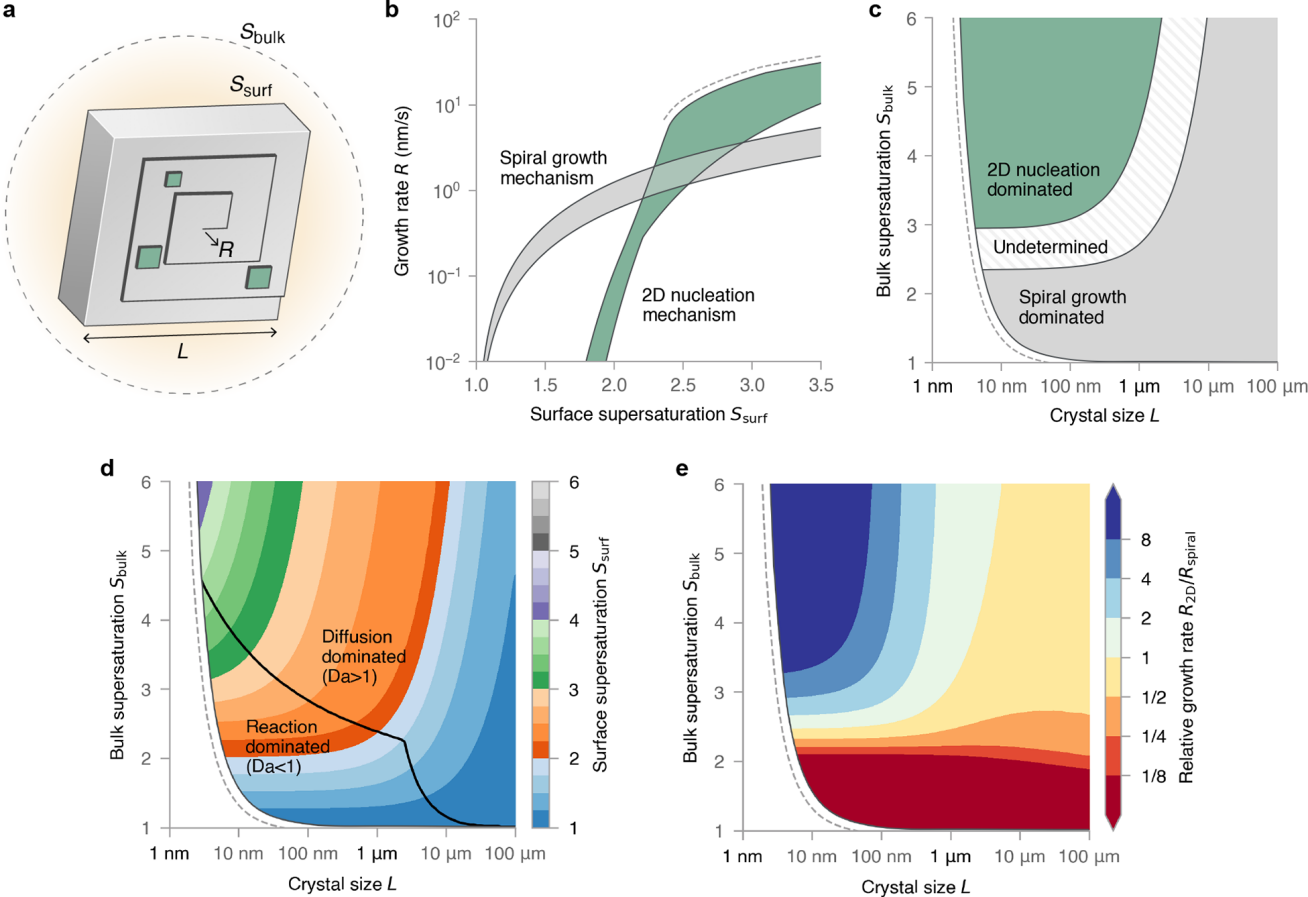
(a) Illustration of a calcite crystal growing via 2D nucleation (green islands) and a spiral hillock. The surface supersaturation *S*_surf_, bulk supersaturation *S*_bulk_, crystal size *L*, and normal growth rate *R* are depicted. (b) The surface-controlled growth rate of calcite obtained from theoretical fits to AFM data with mass transport accounted for. The shaded areas represent uncertainty in the models. (c) Dominant growth mechanism derived by plugging the surface-controlled kinetics into a model of solute diffusion. (d) *S*_surf_ as a function of *L* and *S*_bulk_. The black line corresponds to the Damköhler number *Da* = 1. We had to estimate some model parameters to produce this plot, see Section 4.1.2. (e) Relative growth rate of 2D nucleation and spiral growth. The growth rates are very similar for *S*_bulk_ ≳ 2.5 and *L* ≳ 1 μm because the growth is diffusion-dominated. (b–e) The dashed lines show the predictive limits of the model.

The growth kinetics of calcite depend on the supersaturation
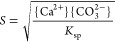
1where {*x*} is the activity of species *x* and *K*_sp_ is the solubility product of calcite. We will use subscripts to distinguish the supersaturation at the crystal surface, *S*_surf_, from the bulk solution, *S*_bulk_ (see Figure 1a).

The kinetics of spiral growth for calcite have previously been measured as a function of *S*_bulk_ using flow-through AFM.^12^ The same study also reported growth by 2D nucleation at *S*_bulk_ = 1.5. However, the surface-controlled kinetics of a crystal must be characterized by the supersaturation at the crystal surface, *S*_surf_. To compute the growth kinetics for both spiral growth and 2D nucleation from these same AFM data,^12^ but as a function of *S*_surf_, we used the critical step length indicator^22^ to determine *S*_surf_ and classical growth theory to extrapolate the results to a wider range of supersaturations. Full details can be found in Section 4.1.

The resulting growth rates are shown in Figure 1b as a function of *S*_surf_, where the shaded areas represent uncertainty in the model. At low supersaturations (*S*_surf_ ≲ 2.2), spiral growth is faster because 2D nucleation is hindered by a large activation barrier. At higher supersaturations (*S*_surf_ ≳ 3.0), 2D nucleation is faster. We cannot determine the faster mechanism in the intervening supersaturations (2.2 ≲ *S*_surf_ ≲ 3.0) due to the uncertainty in the model. The reason 2D nucleation was observed under AFM at *S*_surf_ < *S*_bulk_ = 1.5, in apparent conflict with our model (Figure 1b), is because the crystal surface had not achieved a steady state in the experiment: the expanding spiral hillocks had not been allowed sufficient time to reach the probed region of the surface.

Since step kinetics are sensitive to the solution conditions,^16^ the kinetic curves in Figure 1b will be specific to the solution conditions in the AFM experiment (stoichiometry of {Ca}/{CO_3_} = 1.04 ± 0.01, ionic strength fixed between 0.105 and 0.111 M using NaCl, and a pH of 8.50 maintained with a NaOH buffer). However, the relative speed of 2D nucleation and spiral growth will depend on the relative step density of these two modes, which is principally a function of the supersaturation. The supersaturation reported here that marks the transition between the two growth modes is therefore expected to hold over a much wider range of solution conditions.

### 2.2 Crystals of Finite Size

Since the surface supersaturation *S*_surf_ of a crystal is difficult to measure experimentally and is therefore seldom known, it can be more useful to characterize the growth kinetics in terms of the bulk supersaturation *S*_bulk_ and the crystal size *L*. To this end, we combined the surface-controlled kinetics from the previous section with a simple model of solute diffusion to determine the growth kinetics as a function of the pair (*L*, *S*_bulk_). Full details can be found in Section 4.2.

According to this model, 2D nucleation only dominates over spiral growth when the bulk supersaturation is high (*S*_bulk_ ≳ 3) and the crystal size is small (*L* ≲ 1 μm), see Figure 1c. Significantly, spiral growth is predicted to dominate over 2D nucleation for crystals larger than ∼1 μm, irrespective of the bulk supersaturation. At the super-micron length scale, slow boundary layer diffusion creates a low *S*_surf_ that favors spiral growth. These predictions (Figure 1c) are supported by experimental evidence: intrasectoral zoning, which is a signature of spiral growth,^23,24^ has been observed in calcite crystals for (*L* ∼10 μm, *S*_bulk_ = 3.9)^11^ and (*L* ∼100 μm, *S*_bulk_ = 4.5).^25^

The surface supersaturation surrounding a calcite crystal is shown in Figure 1d as a function of *L* and *S*_bulk_ (we had to estimate some model parameters to produce this plot). The black line in Figure 1d divides the plot into two regimes where growth is limited mainly by (i) surface reaction or (ii) boundary layer diffusion. Strictly speaking, crystal growth is always limited by the surface reaction; however, the surface reaction depends on the surface supersaturation, which is limited by diffusion. We say that growth is limited mainly by diffusion when the Damköhler number *Da* > 1 (see Section 4.2). In the diffusion-dominated regime, *S*_surf_ has a weak dependence on *S*_bulk_ due to a feedback mechanism that attenuates any change in *S*_surf_. Specifically, an increase in *S*_surf_ produces faster crystal growth that acts to decrease *S*_surf_. For this same reason, the crystal growth rate has a weak dependence on the growth mechanism in most of the diffusion-dominated regime; the relative growth rate of 2D nucleation and spiral growth varies by less than a factor of 2 when *L* ≳ 1 μm and *S*_bulk_ ≳ 2.5 (Figure 1e).

**Figure 2:**
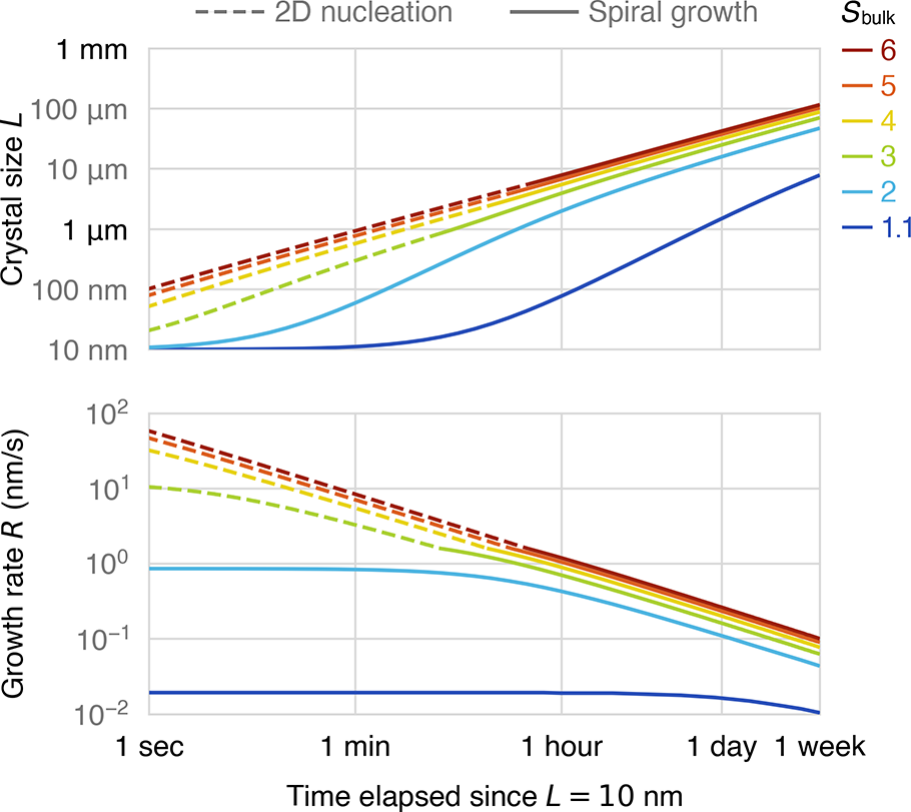
Time evolution of the size, growth rate, and growth mechanism of a single crystal of calcite in a solution with a constant bulk supersaturation, starting from a size *L* = 10 nm.

Figure 2 summarizes the above discussion by showing the predicted time evolution of a single crystal in a solution with a constant bulk supersaturation. As the crystal grows, the growth rate decreases due to increasingly slow boundary layer diffusion. When *S*_bulk_ ≳ 3, the growth rate depends only weakly on *S*_bulk_, and the growth mechanism undergoes a transition from 2D nucleation to spiral growth as *L* exceeds ∼1 μm. It would take more than 1 week for the crystal to reach 100 μm in size when *S*_bulk_ = 3, whereas it would take a matter of hours if growth was always reaction-limited.

### 2.3 Additives Can Change the Dominant Growth Mechanism

In pure solution, 2D nucleation is predicted to dominate over spiral growth only when the supersaturation is high (*S*_bulk_ ≳ 3) and the crystal size is small (*L* ≲ 1 μm). This account, however, is not necessarily accurate when additives are introduced to the solution. We identify four ways that an additive might change the dominant growth mechanism.

**Figure 3:**
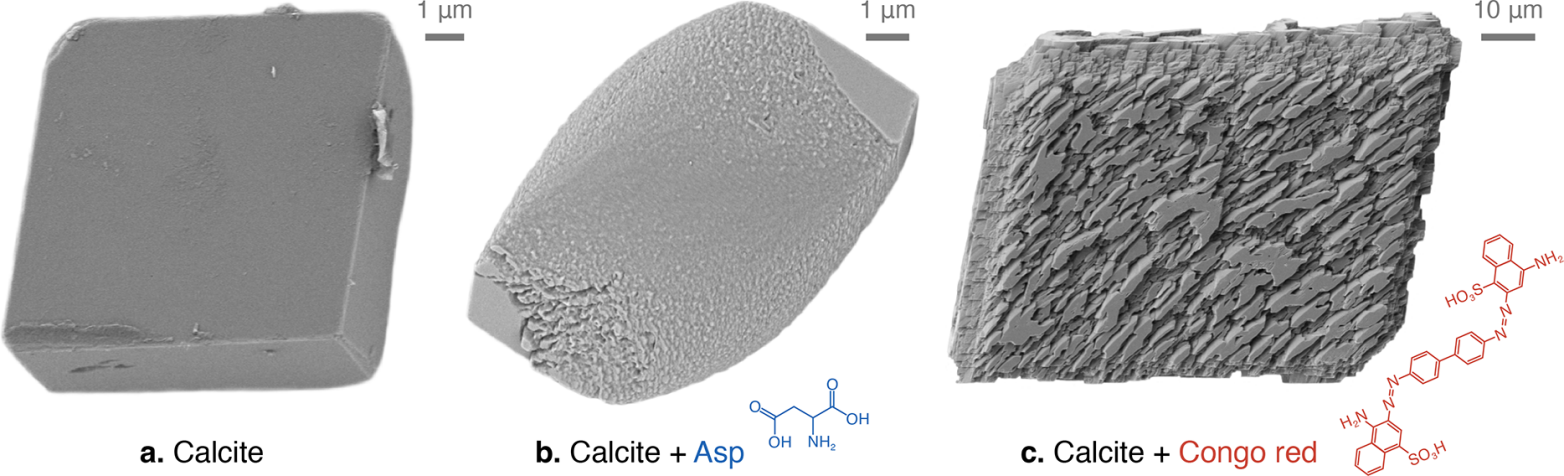
Scanning electron micrographs of three synthetic calcite crystals. (a) The equilibrium morphology in pure solution is a rhombohedron composed of {10.4} faces. (b) Aspartic acid can change the morphology by creating pseudo-faces. These pseudo-faces can engulf the dislocation sources, forcing the residual {10.4} faces to grow by 2D nucleation. (c) Seeded calcite growth in the presence of Congo red produces strongly partitioned surfaces. Growth must occur via 2D nucleation since a dislocation in one partition would be unable to supply steps to its neighboring partitions.

First, an additive could immobilize the screw dislocations by changing the crystal morphology. Calcite has a rhombohedral morphology in pure solution (Figure 3a), but additives can cause steps to pile up to form pseudo-faces.^26^ By increasing the additive concentration, pseudo-faces can become expressed in the morphology to an almost arbitrary degree; for example, very little remains of the {10.4} faces when calcite is precipitated in the presence of a high concentration of Asp (Figure 3b). Since a pseudo-face must engulf any dislocation in its way, the residual {10.4} faces might be free of dislocations and have no option but to grow by 2D nucleation.

Second, an additive could isolate the dislocation sources by partitioning the surface. Poorly soluble additives may aggregate on the crystal surface to form assemblies that are too large to be overgrown.^6,27^ By blocking growth, the assemblies partition the surface such that any dislocation source in one partition would be unable to supply steps to the neighboring partitions, forcing them to grow by 2D nucleation. To illustrate this partitioning effect, Figure 3c shows a calcite crystal overgrown in a solution containing the dye Congo red.

Third, the additive could enhance the 2D nucleation rate, e.g., by providing a site for heterogeneous nucleation, thus shifting the kinetics in favor of 2D nucleation. This effect has not been observed in calcite to the best of our knowledge.

Fourth, imagine a crystal in pure solution within the diffusion-dominated regime. If an inhibitor was introduced to slow down the crystal growth, then *S*_surf_ would increase, shifting the kinetics in favor of 2D nucleation. For example, if *S*_bulk_ = 4, then a crystal will transition from 2D nucleation to spiral growth at a crystal size of approximately 1 μm in pure solution. If, however, the solution included an inhibitor that slowed crystal growth by 50%, then spiral growth would begin to dominate at approximately 2 μm. At 90% inhibition, the transition would occur at approximately 10 μm. In this way, an additive could have a significant effect on the dominant growth mechanism but only at very high inhibitions.

In all four cases, the additive promotes 2D nucleation over spiral growth. Note that, if *L* ≳ 1 μm and *S*_bulk_ ≳ 2.5, then an additive-induced transition from spiral growth to 2D nucleation might not produce a significant change in the overall growth rate since the growth is diffusion-limited (see Figure 1e).

## 3 Conclusions

Boundary layer diffusion can play a critical role in determining the growth rate and growth mechanism of calcite. Additives can complicate the story by immobilizing growth spirals or by reducing the significance of diffusion through growth inhibition. Diffusion might therefore be an important and under-investigated aspect of how additives and even confinement^28^ control the growth and polymorphism of CaCO_3_.

## 4 Methods

### 4.1 Surface-Controlled Kinetics

Calcite growth kinetics have previously been measured as a function of *S*_bulk_ using flow-through AFM.^12^ In this section, we recast those AFM-derived kinetics as a function of *S*_surf_.

#### 4.1.1 Spiral Growth

The step velocities (*v*^±^) and terrace widths (λ^±^) of the acute (−) and obtuse (+) steps of calcite are reported in Table 2 of ref (12) as a function of *S*_bulk_. For each of these measurements, the critical step length can be determined and used as an indicator of the surface supersaturation to establish the true surface-controlled kinetics. We summarize this procedure here. The reader will find a more detailed account elsewhere.^22^

The average critical length can be computed from the experimental step velocities and terrace widths
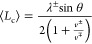
2where
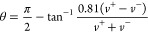
3measures the angle between adjacent spiral turns. From ⟨*L*_c_⟩, the obtuse critical length *L*_c_ follows from the empirical relationship^13^
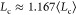
4If the step free energy ϕ was known, then the surface supersaturation *S*_surf_ could be determined from *L*_c_
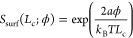
5

In this way, each experimental measurement of the step velocity (*S*_bulk_, *v*) could be mapped to its surface-controlled analogue (*S*_surf_, *v*). The acute and obtuse step velocities *v*^±^(*S*_surf_; ϕ) could then be obtained through linear fits to these new surface-controlled measurements (*S*_surf_, *v*), where the obtuse step velocity is fitted subject to the constraint *v*^+^(*S*_surf_ = 1; ϕ) = 0.

Finally, the normal growth rate of the spiral hillock is
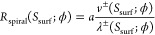
6where *v*^±^(*S*_surf_; ϕ) refers to the linear fits and λ^±^(*S*_surf_; ϕ) is evaluated using eqs 2–5. The step free energy ϕ is estimated to fall in the range of 2.5 ≤ ϕ/*k*_B_*T* ≤ 3.5.^22^ Sampling this range of ϕ produces the range of growth rates shown in Figure 1b.

The role of mass transport in the AFM measurements can be characterized by a boundary layer thickness δ. Since the normal growth rate of the surface *R*(*S*_surf_)/ω will balance with the net flux of solutes across the boundary layer, *D*γ^–1^*K*_sp_^1/2^(*S*_bulk_ – *S*_surf_)/δ, we get
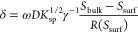
7where ω is the molar volume of calcite, *D* is the ion diffusion coefficient, *K*_sp_ is the solubility product of calcite, and γ is the activity coefficient. If the crystal surface structure has fully reconstructed to achieve a steady state, then *R* in eq 7 will correspond to *R*_spiral_(*S*_surf_; ϕ) in eq 6. In ref (22), three AFM measurements were identified as corresponding to a fully reconstructed surface: (*S*_bulk_, *L*_c_) = (1.674,14.64 nm), (1.819,12.99 nm), and (2.036,12.28 nm). From these three data points, it is possible to determine δ for any given ϕ: If δ was known, then eq 7 could be solved numerically to obtain *S*_surf_ for a given *S*_bulk_, and *L*_c_ would then follow from *S*_surf_ via eq 5. In this way, the value of δ can be optimized for a given ϕ to produce a least squares fit to the three (*S*_bulk_, *L*_c_) data points above.

#### 4.1.2 2D Nucleation

The kinetics of a calcite surface growing by 2D nucleation, *R*_2D_, has never been recorded. However, in the AFM study analyzed in the previous section, a single in situ micrograph (Figure 3c of ref (12)) was presented showing a calcite surface growing by 2D nucleation at *S*_bulk_ = 1.5. The growth rate of the crystal surface in this micrograph, *R*_2D_^*^, can be determined using the step velocities and boundary layer thickness established in the previous section. We will use an asterisk, e.g., *R*_2D_^*^, to denote any quantity that is specific to the micrograph.

We measured the average dimensionless step density in the micrograph to be ρ* = 0.00039. This is equivalent to an average step spacing of 821 nm. If the surface supersaturation *S*_surf_^*^ was known, then *R*_2D_^*^ could be computed with an accuracy dependent on how well ϕ is known
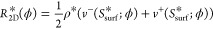
8where *v*^±^(*S*_surf_; ϕ) are the linear fits from the previous section. It follows from eq 7 that
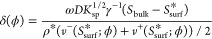
9where the boundary layer thickness δ(ϕ) was established in the previous section to characterize the mass transport in the system. Equation 9 can be solved numerically to find *S*_surf_^*^, and *R*_2D_^*^ then follows from eq 8.

This datum (*S*_surf_^*^(ϕ), *R*_2D_^*^(ϕ)) can be extrapolated to other surface supersaturations using classical nucleation theory^29^
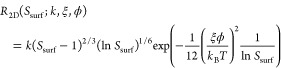
10where *k* is a kinetic coefficient and ξ is a shape factor defined as the ratio of the perimeter of the critical nucleus to the square root of its area. We sample the same step free energy range as in the previous section, 2.5 ≤ ϕ/*k*_B_*T* ≤ 3.5, and assume the shape factor to fall between that of a circle () and that of a calcite rhombohedron (). The shape of the post-critical islands in the AFM micrograph fell within this range (we measured ξ* ≈ 3.8). For each combination of (ξ, ϕ), the prefactor *k* is fixed by the datum (*S*_surf_^*^(ϕ), *R*_2D_^*^(ϕ)) according to eq 10.

The classical nucleation theory that we use to extrapolate the results (eq 10) implicitly assumes that islands only interact with other islands after exceeding the critical size. This is not possible when the supersaturation is so high that the average step spacing (*v*^–^ + *v*^+^)/(2*R*_2D_) is comparable to the critical nucleus size *L*_c_ = 2ϕ/(*k*_B_*T* ln *S*_surf_). Therefore, in constructing Figure 1b, we only explored *S*_surf_ for each parameter set (*k*, ξ, ϕ) up until (*v*^–^ + *v*^+^)/(2*R*_2D_) = *L*_c_. Furthermore, in constructing Figures 1d,e and 2, we estimated ϕ = 3*k*_B_*T* and ξ = 3.8, and we only explored crystal sizes *L* > 2*L*_c_ as it is unclear what the spiral kinetics would be for smaller crystals.

### 4.2 Transform (*L*, *S*_bulk_) to *S*_surf_

The previous section derived the growth kinetics of calcite as a function of surface supersaturation *S*_surf_. The kinetics can alternatively be characterized as a function of crystal size *L* and bulk supersaturation *S*_bulk_.

In the steady state, the supersaturation field surrounding a crystal will satisfy the diffusion equation ∇^2^*S* = 0 subject to two boundary conditions: (i) the solute flux −*D* ∇ *S* must balance with the growth rate *R* of the crystal at each point on the surface and (ii) *S* = *S*_bulk_ in the far-field. To solve this equation analytically, the rhombohedral calcite crystal of length *L* can be approximated as a sphere with an equivalent surface area, i.e., with a radius . In this case, the diffusion equation solves to
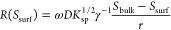
11

This equation can be solved numerically to find *S*_surf_ for any *L*, *S*_bulk_, and any growth mechanism encapsulated in *R*. Setting *R* = max (*R*_spiral_, *R*_2D_) captures the faster of the two growth modes. From *S*_surf_, the growth kinetics and dominant growth mechanism can be identified as before.

Within this model, the relative significance of surface reaction and solute diffusion can be quantified by the Damköhler number, *Da*. This quantity is defined as the ratio of the crystal growth rate under reaction control to the crystal growth rate under diffusion control,
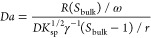
12

When *Da* ≪ 1, crystal growth is limited by the surface reaction, and when *Da* ≫ 1, growth is limited by diffusion. The curve *Da* = 1 therefore divides these two growth regimes, as shown in Figure 1d.

### 4.3 Calcite Crystal Examples

Figure 3 features three synthetic calcite crystals grown from three different solutions.

Figure 3a shows a calcite crystal grown from pure solution using the ammonium diffusion method.^30^ Aqueous solution containing [CaCl_2_] = 10 mM was placed in a plastic Petri dish containing a glass slide. The dish was covered with a perforated Parafilm and placed for 2 days in a desiccator previously charged with 5 g of freshly crushed (NH_4_)_2_CO_3_ powder. Full details can be found elsewhere.^2^

Figure 3b shows a calcite crystal grown using the above method except the solution also contained [Asp] = 50 mM.

Figure 3c shows a calcite crystal that was initially a ∼50 μm seed. It was subsequently overgrown for 3 days in a solution comprising [CaCl_2_] = [NaHCO_3_] = 10 mM and [Congo red] = 20 μM. Full details can be found elsewhere.^6^
